# Mortality among healthcare workers in Indonesia during 18 months of COVID-19

**DOI:** 10.1371/journal.pgph.0000893

**Published:** 2022-12-09

**Authors:** Lenny L. Ekawati, Ahmad Arif, Irma Hidayana, Ahmad Nurhasim, M. Zakiyuddin Munziri, Karina D. Lestari, Amanda Tan, Firdaus Ferdiansyah, Fikry Nashiruddin, Qorinah E. S. Adnani, Halik Malik, Tri Maharani, Andy Riza, Monalisa Pasaribu, Khairul Abidin, Adhi A. Andrianto, Nursalam Nursalam, A. V. Sri Suhardiningsih, Ade Jubaedah, N. S. Widodo, Henry Surendra, Herawati Sudoyo, Adrian D. Smith, Philip Kreager, J. Kevin Baird, Iqbal R. F. Elyazar

**Affiliations:** 1 Oxford University Clinical Research Unit (OUCRU), Jakarta, Indonesia; 2 Nuffield Department of Medicine, University of Oxford, Oxford, United Kingdom; 3 LaporCOVID-19, Jakarta, Indonesia; 4 St. Lawrence University, Canton, New York, United States of America; 5 The Conversation, Jakarta, Indonesia; 6 Faculty of Medicine, Padjadjaran University, Bandung, Indonesia; 7 Indonesian Doctor Association, Jakarta, Indonesia; 8 National Institute of Health Research and Development, Ministry of Health, the Republic of Indonesia, Jakarta, Indonesia; 9 Indonesia National Nurses Association of East Java Province, Surabaya, Indonesia; 10 Faculty of Nursing, Airlangga University, Surabaya, Indonesia; 11 Indonesian Midwives Association, Jakarta, Indonesia; 12 Association of Indonesian Medical Laboratory Technologist, Jakarta, Indonesia; 13 Mochtar Riady Institute for Nanotechnology, Tangerang, Banten, Indonesia; 14 Nuffield Department of Population Health, University of Oxford, Oxford, United Kingdom; 15 Institute of Human Sciences, University of Oxford, Oxford, United Kingdom; University of Embu, KENYA

## Abstract

The impact of SARS-CoV-2 infections upon Indonesian health care workers (HCWs) is unknown due to the lack of systematic collection and analysis of mortality data specific to HCWs in this setting. This report details the results of a systematic compilation, abstraction and analysis of HCW fatalities in Indonesia during the first 18 months of COVID-19. HCW who passed away between March 2020 and July 2021 were identified using *Pusara Digital*, a community-based digital cemetery database dedicated to HCW. We calculated the mortality rates and death risk ratio of HCWs versus the general population. The analysis indicates that at least 1,545 HCWs died during the study period. Death rates among males and females HCWs were nearly equivalent (51% vs. 49%). The majority were physicians and specialists (535, 35%), nurses (428, 28%), and midwives (359, 23%). Most deaths occurred between the ages of 40 to 59 years old, with the median age being 50 years (IQR: 39–59). At least 322 deaths (21%) occurred with pre-existing conditions, including 45 pregnant women. During the first 18 months of COVID-19 in Indonesia, we estimated a minimum HCW mortality rate of 1.707 deaths per 1,000 HCWs. The provincial rates of HCW mortality ranged from 0.136 (West Sulawesi) to 5.32 HCW deaths per 1,000 HCWs (East Java). The HCW mortality rate was significantly higher than that of the general population (RR = 4.92, 95% CI 4.67–5.17). The COVID-19 pandemic in Indonesia resulted in the loss of many hundreds of HCWs, the majority of whom were senior healthcare workers. The HCW mortality rate is five times that of the general population. A national systematic surveillance of occupational mortality is urgently needed in this setting.

## Introduction

The disease resulting from over 500 million confirmed SARS-CoV-2 infections (COVID-19) had caused approximately 6.3 million deaths globally [1]. Healthcare workers (HCWs) serving these patients are at high risk of becoming infected [2, 3]. In the early months of the COVID-19 pandemic, a survey of morbidity and mortality on HCWs in 130 countries counted hundreds of deaths [4]. After 18 months, among 135 million HCWs, World Health Organization (WHO) estimated at least 115,000 (range: 80,000–180,000) HCWs died as a consequence of SARS-CoV-2 infection [5].

The first cluster of SARS-CoV-2 infections in Indonesia was detected in early March 2020 [6]. Later in mid-2021, the Delta variant of SARS-CoV-2 dominated the surge [7, 8], and overwhelmed hospital capacities across Indonesia [8]. The surge in Delta variant cases prompted stricter emergency restrictions on people’s movement and gathering [9]. SARS-CoV-2 test positivity rates during this crisis period approached 40% [9]. The rapid rise of hospitalizations, scarcity of supplies, and ICU bed needs outstripping availability have caused HCW anxiety and exhaustion [10].

The high proportion of COVID-19 cases among HCWs working in patient ward was identified [11, 12], and their risk of contracting COVID-19 is higher than the general population [13]. Logic evidence informs safe standard operating procedures (SOPs) in healthcare settings [14], personal protective equipment (PPE) inspection, and HCWs practice review are critically important to avoid negligence in caring for infectious COVID-19 patients [15–17]. Inadequate PPE, or being unfamiliar with wearing it has contributed to a substantially elevated risk of COVID-19 compared to other groups not working in a hospital setting [18–20]. Working environment, type of occupation, length of exposure with COVID-19 patients, and testing availability have been identified as factors associated with SARS-CoV-2 infection among HCWs [20–23].

Heavy burden in managing COVID-19 patients may lead to emotional exhaustion and jeopardize professional efficacy [24]. However, a manageable working schedule, mental health, psychosocial support, remuneration and incentives are required for HCWs’ welfare [25–27]. Safety issues and high protective working conditions for pregnant HCWs and those with higher vulnerability due to age, ethnicity, social determinants, or underlying conditions should be implemented and carefully monitored [28].

The Indonesian Digital Cemetery (*Pusara Digital*; https://nakes.laporcovid19.org/) by far is the most comprehensive source of data on HCW mortality in Indonesia. *Pusara Digital* received and recorded mortality data as both public service and memorial to those lives lost in humanitarian service. The study represents an effort to utilize that resource and provides a descriptive analysis of mortality due to COVID-19 among HCWs during 2020–2021. This report derives a systematic quantitative estimate of COVID-19 mortality among HCWs in Indonesia, and its comparison with general population.

## Methods

### Source of routine information

The daily verified COVID-19 confirmed cases and death data from 34 provinces were available on the government website, managed by the Center of Health Data of the Indonesian Ministry of Health (MoH) [29]. The research team extracted the daily provincial-level aggregated data recorded between March 2020 and July 2021, then entered the extracted data into an Excel database. The number of population and the number of HCW by provinces were extracted from the 2020 Indonesia Annual Health Profile [30].

### Collection of demographic and health information for HCW deaths

The research team downloaded HCW mortality data from Pusara Digital, a web-based digital cemetery database dedicated to HCW’s. The platform is operated by LaporCOVID-19, a registered not-for-profit organization established in March 2020, by public health experts, scientists, journalists, and social activists committed to the evidence-based and unbiased characterization of the social economic, and health impacts of COVID-19 on the Indonesian people.

LaporCOVID-19 organized 35 volunteers to manage *Pusara Digital* website in September 2020 and has undertaken two methods of HCW mortality data searching. First, the volunteers looked for daily obituaries on online news and social media (Twitter, Facebook, Instagram). Second, they looked for daily obituaries on online news and social media (Twitter, Facebook, Instagram). From those sources, the volunteers curated essential variables and organized testimonials from surviving family and colleagues that were sent to the website.

Data were entered into an Excel database with limited access and password protected facility. The variables consisted of full name, title, age, gender, education, healthcare work type, work setting, latitude and longitude of district/city, underlying health condition, date of birth and death, and COVID-19 diagnostic status (suspect, probable, or confirmed) defined by the Indonesian MoH [31]. All individual information of the deceased is treated as confidential. After January 2021, when the government of Indonesia rolled out the COVID-19 vaccination, the team sought and recorded the official vaccination status of each deceased (dates and type of vaccination by, and vaccine’s name). HCWs were afforded top priority in that roll out scheme [32].

### Statistical analysis

The frequency of HCW mortality was cross-tabulated by province, island, gender, age, occupational type, working place, COVID-19 status at the time of death, COVID-19 patient care, and COVID-19 vaccination status. We calculated mortality rate of the general population by dividing the number of reported deaths by the population at risk during pandemic. HCW mortality rate was calculated by dividing the number of HCW reported deaths and the number of HCW at risk during pandemic. The number of population and the number of HCW by provinces were extracted from the 2020 Indonesia Annual Health Profile [30].

We calculated the death risk ratio to compare the likelihood of the death for HCW group to that of the general population. A 95% confidence interval of risk ratio was calculated using the log-transformation method. Mortality rate and risk ratio analysis was carried out at provincial, island, and national level over time. The statistical correlation between HCW mortality rate and the general population mortality rate was calculated using Spearman’s rank correlation and p-value = 0.05 as a threshold of statistical significance. Data were analyzed using Stata statistical version 12 (StataCorp, College Station, TX, USA). Mapping of provincial mortality rates used QGIS mapping software version 3.16.6 [33].

### Ethics statement

This study was approved by the Health Research Ethics Committee of the National Institute of Health Research and Development, Ministry of Health, Republic of Indonesia (LB.02.01/2/KE/620/2020). The requirement for subject consent was waived as this was a secondary analysis of extracted data from the online media, and report from the professional medical and health associations.

## Results

### HCWs mortality

Throughput the first 18 months of COVID-19 pandemics, *Pusara Digital* documented 1,545 HCW deaths (Table 1). The deaths of males and females slightly differed (51% vs 49%). The median age among lost HCWs was 50 years old (IQR: 39–59 years). The largest age group was 50–59-year-olds (287 individuals, 19% of the total), followed by HCWs less than 40 years old, 40–49-year-olds (223 individuals, 14%), and 30–39 years old (185 individuals, 12%).

**Table 1:** Individual characteristics of deceased healthcare workers between March 2020 and July 2021 in Indonesia.

Characteristics	Sumatra	Java	Bali & Nusa Tenggara	Kalimantan	Sulawesi	Maluku & Papua	Total	Percent
**Total HCW population** ^1^	**232116**	**418970**	**59207**	**65778**	**96992**	**32198**	**905261**	**100%**
**Total HCW deaths** ^2^	**163**	**1208**	**29**	**68**	**63**	**14**	**1545**	**0.2%**
**Mortality rate per 1,000**	**0.702**	**2.883**	**0.490**	**1.034**	**0.650**	**0.435**	**1.707**	
**Gender**								
Male	86	607	8	43	36	5	785	50.8%
Female	77	601	21	25	27	9	760	49.2%
**Age group (years)**								
20–29	9	52	4	1	4	0	70	4.5%
30–39	14	152	4	7	6	2	185	11.9%
40–49	32	167	5	9	8	2	223	14.4%
50–59	33	223	2	13	12	4	287	18.7%
60–69	18	100	2	5	10	2	136	8.9%
70+	6	79	3	3	7	0	98	6.3%
Missing data	51	435	9	30	16	4	545	35.3%
**Age (Median, IQR)**	50 (42–56)	50 (39–59)	46 (32–59)	50 (42–58)	52 (43–65)	51 (45–55)	50 (39–59)	
**Occupational type**								
Nurses	26	362	6	21	9	3	427	27.6%
Midwives	44	283	10	7	11	4	359	23.2%
General Practitioners	30	228	6	12	17	1	294	19.0%
Specialists	46	166	2	12	13	2	241	15.6%
Pharmacists	5	38	1	5	2	2	53	3.2%
Laboratory Technologists	4	35	4	2	2	2	49	3.2%
Public Health Officers	2	27	0	5	5	0	39	2.5%
Dentists	0	31	0	2	3	0	36	2.3%
Administrative Staff	2	8	0	0	0	0	10	0.6%
Radiographers	1	8	0	1	0	0	10	0.6%
Nutritionists	1	7	0	0	1	0	9	0.6%
Others	2	15	0	1	0	0	18	1.2%
**COVID-19 status**								
Positive	149	1137	26	61	59	11	1443	93.4%
Probable	14	71	3	7	4	3	102	6.6%
**Working place**								
Hospital	68	523	12	34	28	5	670	43.4%
Primary Health Center	41	264	4	18	12	5	344	22.3%
Clinical/Private Practice	14	147	3	1	5	0	170	11.0%
Health and Medical School	9	55	3	1	6	1	75	4.9%
Other	31	219	7	14	12	3	286	18.5%
**COVID-19 patient care**								
Yes	72	484	7	29	21	3	616	39.9%
Probable	38	345	8	14	17	5	427	27.6%
No	53	379	14	25	25	6	502	32.5%
**Period of death and vaccination status**								
Pre-vaccination campaign	90	499	15	41	35	6	686	44.4%
During vaccination campaign	1	3	0	0	1	1	5	0.3%
Post-vaccination campaign								
• Vaccinated	4	28	0	0	2	0	34	2.2%
• Unvaccinated	10	56	4	4	1	2	77	5.0%
• Unavailable vaccination status	58	622	10	22	25	6	743	48.1%
**Comorbid**								
Yes	24	218	4	12	12	7	277	17.9%
No	139	990	25	56	51	7	1268	82.1%
**Total comorbid**								
1	18	144	3	10	9	5	189	68.2%
2	6	54	1	1	3	2	67	24.2%
≥3	0	20	0	1	0	0	21	7.6%
**Comorbid type**								
Asthma	0	16	0	1	0	0	17	6.1%
Autoimmune disorder	0	3	0	1	0	0	4	1.4%
Cancer	2	5	0	0	0	1	8	2.9%
Cardiovascular disease	2	22	0	1	0	1	26	9.4%
Dengue fever	0	2	0	0	0	0	2	0.7%
Diabetes mellitus	9	104	1	3	5	2	124	44.8%
Gastrointestinal disease	1	1	0	0	0	0	2	0.7%
Haematological disorder	0	1	0	0	0	0	1	0.4%
Hepatitis	0	2	0	0	0	0	2	0.7%
Hernia nucleus pulposus	0	1	0	0	0	0	1	0.4%
HIV	0	0	0	0	1	0	1	0.4%
Hypertension	3	22	0	3	1	2	31	11.2%
Hyperthyroid	0	2	0	0	0	0	2	0.7%
Kidney disease	0	4	0	0	1	0	5	1.8%
Neurological disorder	0	1	0	0	0	0	1	0.4%
Obesity	0	7	0	0	1	1	9	3.2%
Parkinson disease	0	1	0	0	0	0	1	0.4%
Pneumonia	0	2	1	0	0	0	3	1.1%
Pre-eclampsia	1	0	0	0	0	0	1	0.4%
Respiratory disease	1	1	0	0	0	0	2	0.7%
Stroke	0	1	0	0	0	0	1	0.4%
Thyroid	1	0	0	0	0	0	1	0.4%
Typhus	1	1	0	0	0	0	2	0.7%
Unknown	3	19	2	3	3	0	30	10.8%

Data sources:

^1^The Indonesian Ministry of Health, as per December 31, 2020, http://bppsdmk.kemkes.go.id/info_sdmk/info/#

^2^Pusara Digital LaporCOVID-19, as per July 31, 2021, https://nakes.laporcovid19.org/

Among 1,545 HCW deaths, 535 (35%) were general practitioners and specialized medical practitioners, 428 (28%) were nurses, 359 (23%) midwives, 50 (3%) pharmacists, 49 (3%) medical laboratory technologists, and 36 (2%) dentists (Table 1). There were 1,443 (93%) HCWs deaths with confirmed positive COVID-19 infection, while the remaining 102 (7%) were categorized as probable COVID-19 deaths. The underlying health conditions recorded at 277 (18%) HCWs were diabetes mellitus (45%), hypertension (11%), cardiac disease (9%), asthma (6%), cancers (3%), and obesity (3%). Among those with comorbidities, 189 (62%) deceased of HCWs had a single comorbid condition, while 67 (24%) and 21 (8%) HCWs had two or more comorbid conditions.

Forty-five female HCWs (5.9%) were known to have been pregnant prior to their deaths. Sixteen delivered preterm infants immediately prior to their deaths, and 29 women died while pregnant. Nine (18%) of them died before the vaccination program was implemented, while 35 (78%) female HCWs did not receive the vaccine [34] (range time of death: March 19^th^ to July 30^th^, 2021). Only one pregnant HCW who died in May 2021 had received the first dose.

This study included HCW deaths at all healthcare delivery settings, including hospitals (670, 43%), primary health centers (344, 22%), private practices and health clinics (170, 11%), health and medical school (75, 5%), and others (286, 19%). Approximately 39.9% of the deceased were directly involved in routine COVID-19 patient management, while over half may have been occasionally on the COVID-19 wards (27.6%) or were not assigned (32.5%) in the COVID-19 ward.

**Fig 1:**
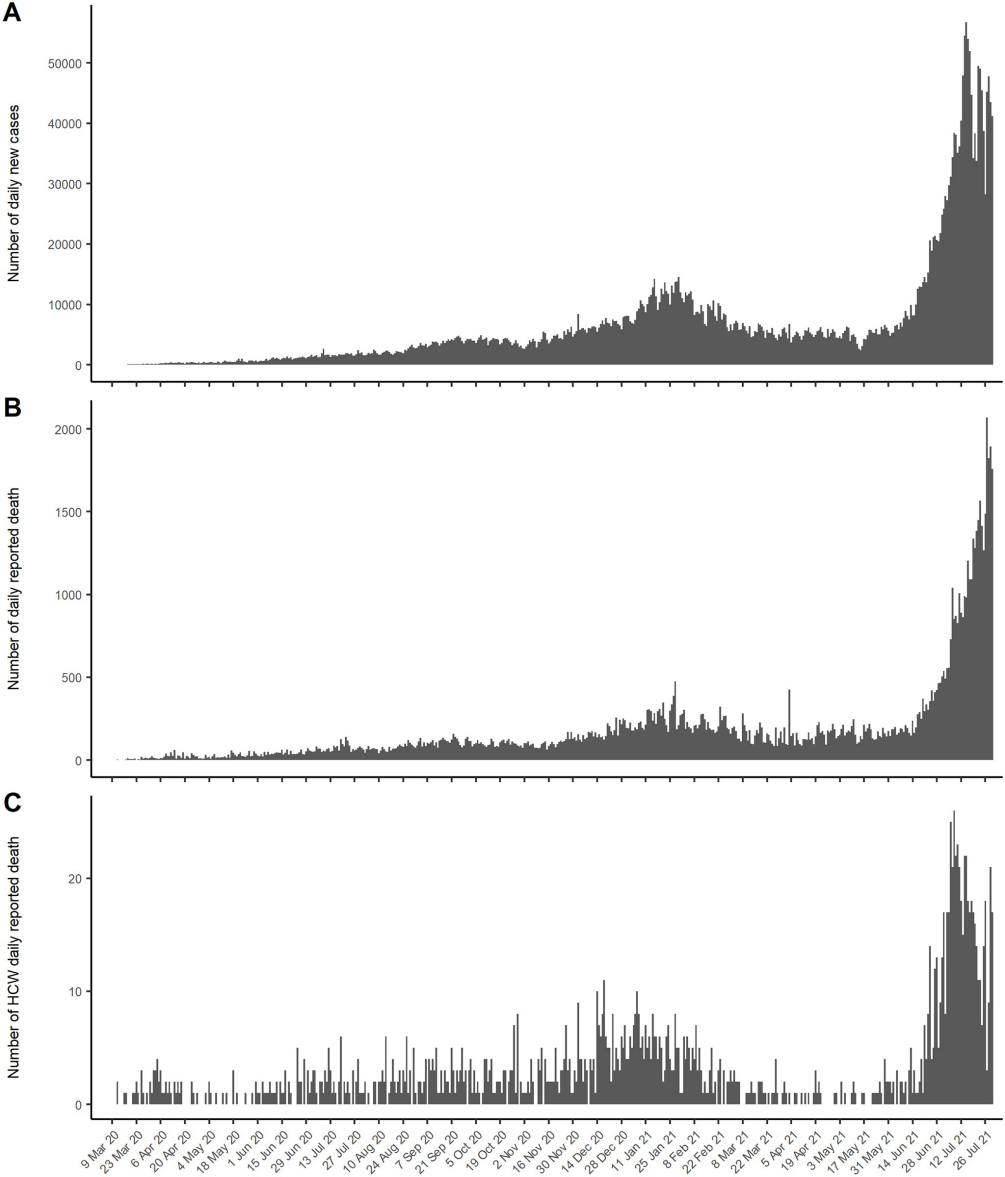


The first HCW deaths were recorded on March 12^th^, 2020, just ten days after the first confirmed COVID-19 cluster was identified in Indonesia. Fig 1C depicts the daily number of HCW fatalities. The days with the most HCW death were December 18th, 2020 (11) and January 6th, 2021 (10), with an average of five HCWs dates per day (range: 0–11 individuals). From the final days of February 2021, the number of late HCW steeply plummeted and continued to decline until May 2021 with a total of 25 deaths. Nonetheless, dramatic leaps occurred from mid-June to July 2021, when the highest daily fallen HCWs accounted for 14 individuals on June 24, 2021 and 26 individuals on July 8, 2021. The average death per day was 10 HCWs, ranging from 0–26 individuals.

A vaccination campaign for health care workers was initiated since mid-January 2021. A month later, the Indonesian Ministry of Health reported that 85% of targeted HCWs were vaccinated, excluding those with comorbid conditions or who were pregnant [35, 36]. *Pusara Digital* recorded more than one-thirds of all HCW deaths prior to the launch of the vaccination program (Table 1). We were only able to obtain the vaccination status of 116 (7.5%) HCWs due to the fact that the vaccination status of nearly half of the deceased was not publicly available. According to the available data, 54 (12%) HCWs involved with COVID-19 patient care and had an underlying health condition.

### Mortality rate of HCW population and the general population

The number of daily new infections first peaked at just under 15,000 during early 2021 (Fig 1A), then steadily declined and leveled at about 6,000 by June 2020. Thereafter, a very substantial surge occurred, reaching a peak of 56,575 cases on July 15^th^, 2021. COVID-19 caused a 10-fold surge in mortality attributable to COVID-19 (2069 deaths on July 27^th^, 2021 vs. typically <200 daily since early 2020, Fig 1B).

Fig 1C indicates the average rate of mortality in the first six months of the COVID-19 pandemic was 0.04/1,000 HCWs and 0.11/1,000 HCWs during the second six months (September 2020 to February 2021). Between March and July 2021, the average of mortality rate was 0.16/1,000 HCWs. Mortality rate in July 2021 was higher than mortality rate from the previous months (0.56/1,000 vs. 0.07/1,000).

Table 2 shows the COVID-19 mortality rate of the HCW population at the national and provincial levels. Between March 2020 and July 2021, the national HCW mortality rate was 1.707 deaths per 1,000 HCWs. On Java Island, the HCW mortality rate was 2.883 deaths per 1,000 HCWs, which was significantly higher than the national HCW mortality rate (p< 0.001). About 46% of HCWs and 60% of the Indonesian population reside on Java Island, which accounts for 71% of all COVID-19 total deaths in Indonesia.

**Table 2:** Mortality rates of HCW and general population in Indonesia between March 2020 and July 2021.

Province/Island	Health workers			General Population^3^			Death Relative Ratio	
	Population^1^	Reported death^2^	Mortality rate (per 1000)	Population	Reported death	Mortality rate (per 1000)	Ratio	95% CI
**Sumatera**								
Aceh	40,110	18	0.449	5,459,891	988	0.181	**2.48**	**1.56–3.95**
North Sumatera	48,841	53	1.085	14,703,532	1,465	0.100	**10.89**	**8.28–14.32**
West Sumatera	22,416	9	0.401	5,498,751	1,496	0.272	1.48	0.77–2.84
Riau	20,717	24	1.158	7,128,305	2,592	0.364	**3.19**	**2.13–4.76**
Jambi	17,636	7	0.397	3,677,894	425	0.116	**3.43**	**1.63–7.25**
South Sumatera	32,666	15	0.459	8,567,923	2,055	0.240	**1.91**	**1.15–3.18**
Bengkulu	11,040	5	0.453	2,019,848	285	0.141	**3.21**	**1.33–7.77**
Lampung	23,912	24	1.004	8,521,201	2,006	0.235	**4.26**	**2.85–6.37**
Bangka Belitung	6,692	1	0.149	1,517,590	663	0.437	0.34	0.05–2.43
Riau Islands	8,086	7	0.866	2,242,198	1,161	0.518	1.67	0.80–3.51
**Total**	**232,116**	**163**	**0.702**	**59,337,133**	**13,136**	**0.221**	**3.17**	**2.72–3.70**
**Java**								
DKI Jakarta	57,439	180	3.134	10,644,986	12,173	1.144	**2.74**	**2.37–3.17**
West Java	106,128	213	2.007	49,935,858	9,363	0.188	**10.70**	**9.35–12.26**
Central Java	105,626	187	1.770	34,940,078	19,343	0.554	**3.20**	**2.77–3.69**
DI Yogyakarta	17,800	19	1.067	3,882,288	3,401	0.876	1.22	0.78–1.91
East Java	106,210	565	5.320	39,886,288	20,330	0.510	**10.44**	**9.60–11.34**
Banten	25,767	44	1.708	13,160,496	1,923	0.146	**11.69**	**8.67–15.75**
**Total**	**418,970**	**1,208**	**2.883**	**152,449,994**	**66,533**	**0.436**	**6.61**	**6.24–6.99**
**Bali and Nusa Tenggara**								
Bali	20,097	13	0.647	4,380,824	2,151	0.491	1.32	0.76–2.27
West Nusa Tenggara	18,458	11	0.596	5,125,622	568	0.111	**5.38**	**2.96–9.76**
East Nusa Tenggara	20,652	5	0.242	5,541,394	725	0.131	1.85	0.77–4.46
**Total**	**59,207**	**29**	**0.490**	**15,047,840**	**3,444**	**0.229**	**2.14**	**1.49–3.08**
**Kalimantan**								
West Kalimantan	16,780	9	0.536	5,134,760	650	0.127	**4.24**	**2.20–8.18**
Central Kalimantan	13,036	9	0.690	2,769,156	861	0.311	**2.22**	**1.15–4.28**
South Kalimantan	17,283	22	1.273	4,303,979	1,351	0.314	**4.06**	**2.53–6.17**
East Kalimantan	14,580	27	1.852	3,793,152	3,345	0.882	**2.10**	**1.44–3.07**
North Kalimantan	4,099	1	0.244	768,505	348	0.453	0.54	0.08–3.83
**Total**	**65,778**	**68**	**1.034**	**16,769,552**	**6,555**	**0.391**	**2.64**	**2.08–3.36**
**Sulawesi**								
North Sulawesi	11,867	5	0.421	2,528,794	704	0.278	1.51	0.63–3.65
Central Sulawesi	16,177	5	0.309	3,096,976	642	0.207	1.49	0.62–3.59
South Sulawesi	40,256	48	1.192	8,928,004	1,338	0.150	**7.96**	**5.97–10.61**
Southeast Sulawesi	15,857	3	0.189	2,755,589	356	0.129	1.46	0.47–4.56
Gorontalo	5,456	1	0.183	1,219,576	228	0.187	0.98	0.14–6.99
West Sulawesi	7,379	1	0.136	1,405,012	170	0.121	1.12	0.16–8.00
**Total**	**96,992**	**63**	**0.650**	**19,933,951**	**3,438**	**0.172**	**3.77**	**2.94–4.83**
**Maluku and Papua**								
Maluku	8,349	4	0.479	1,831,880	226	0.123	**3.88**	**1.45–10.43**
North Maluku	6,693	2	0.299	1,278,764	238	0.186	1.61	0.40–6.46
West Papua	5,593	4	0.715	981,822	285	0.290	2.46	0.92–6.61
Papua	11,563	4	0.346	3,435,430	264	0.077	**4.50**	**1.68–12.08**
**Total**	**32,198**	**14**	**0.435**	**7,527,896**	**1,013**	**0.135**	**3.23**	**1.91–5.47**
**Indonesia**	**905,261**	**1,545**	**1.707**	**271,066,366**	**94,119**	**0.347**	**4.92**	**4.67–5.17**

Data sources:

^1^The number of HCWs (medical doctors, nurse, midwives, pharmacists, laboratory technicians, public health staff) and the number of population at provincial level was extracted from the 2020 Annual Indonesia Health Profile by the Ministry of Health

^2^The number of HCWs death was extracted from the *Pusara Digital*, https://nakes.laporcovid19.org/

^3^The number of general population was extracted from the Emerging Infectious Disease website, Ministry of Health, https://infeksiemerging.kemkes.go.id/dashboard/covid-19

**Fig 2:**
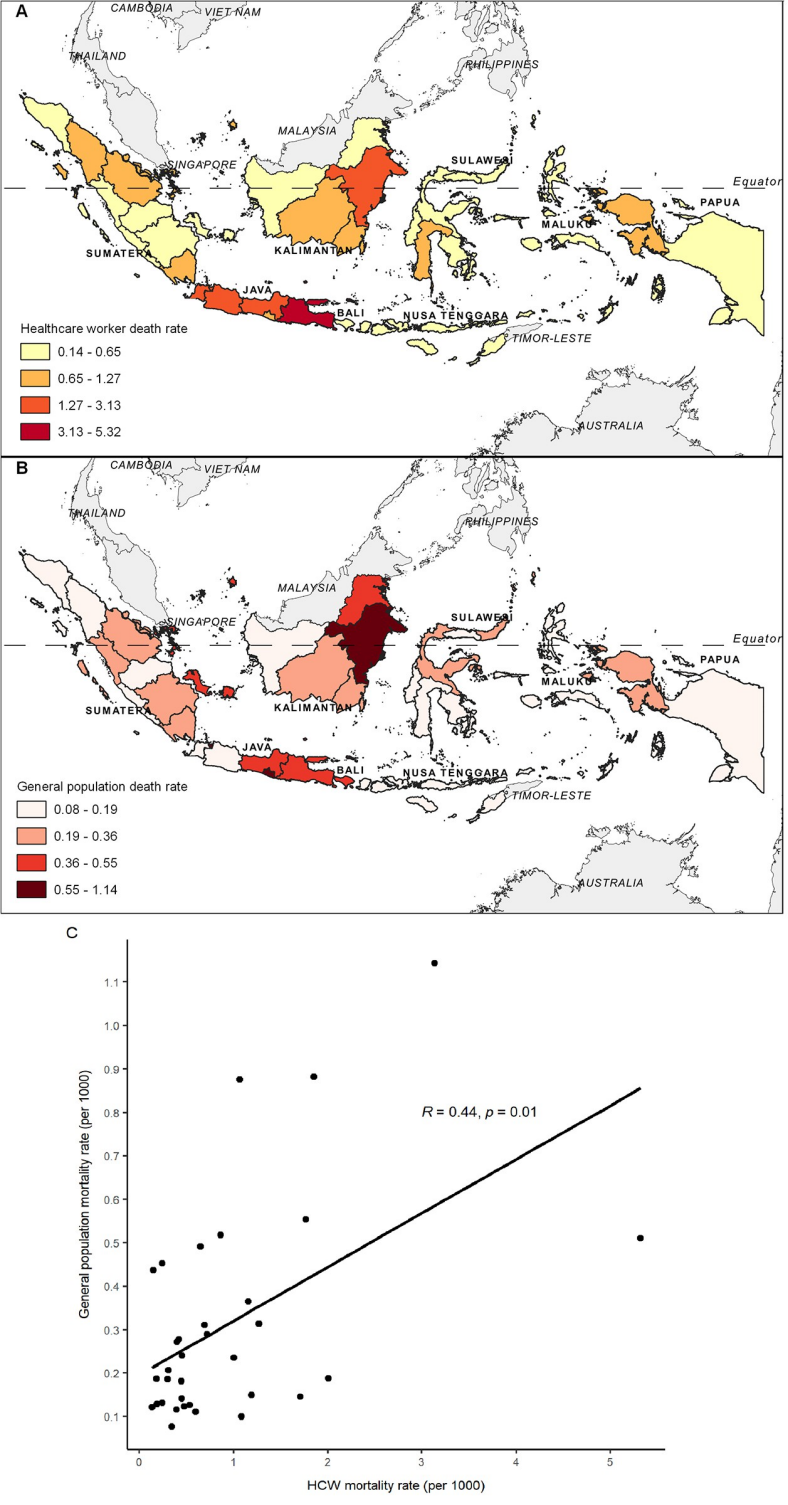


Fig 2A shows a range of provincial HCW mortality rates, from 0.136/1,000 HCWs (West Sulawesi) to 5.319/1,000 HCWs (East Java). Five provinces had higher HCW mortality rates than the national average, including Central Java (1.770); East Kalimantan (1.851); West Java (2.001); Jakarta (3.134); and East Java (5.319).

During the same time period, the COVID-19 mortality rate among the general population was 0.347 deaths per 1,000 people. Fig 2B depicts provincial rates ranging from 0.077/1,000 people (Papua Province) to 1.144/1,000 people (Jakarta Province). The provincial COVID-19 mortality rate among HCW was correlated with that of the general population (Rs = 0.44, p = 0.009) (Fig 2C).

Table 2 shows the death risk ratio between HCWs and the general population. The national death risk ratio for HCW compared to the general population was 4.92 (95% CI 4.67–5.17). In Java, the HCW death risk ratio was significantly higher than that of the general population (RR = 6.61, 95% CI 6.24–6.99). Provincial death risk ratios ranged from 0.34 (95% CI 0.05–2.43; Bangka Belitung Islands) to 11.69 (95% CI 8.67–15.75; Banten).

## Discussion

This first report shows the devastating impact of the COVID-19 pandemic on the Indonesian healthcare workforce. We found at least 1,545 HCWs were lost. The national HCW mortality rate was estimated at 1.707/1,000 HCW deaths. There is no record of confirmed case counts among HCW. However, Indonesian professional healthcare organizations estimated at least 15,000 of their members had been infected by the SARS-CoV-2 virus during 18 months of pandemics [37].

A crude case fatality rate for HCWs is estimated to be five times higher than the general population. If true, this suggests that certain hospital exposure characteristics, such as higher infectious dose, exacerbate the risk of death, e.g., the rapid loss of HCW is alarming due to relatively low numbers of health practitioners, Indonesia’s health services were already stretched even before COVID-19 pandemic, with only two physicians and 15 nurses per 10,000 population in 2019 [30]. In comparison, Philippines served by six physicians and 49 nurses [38], while Malaysia is assisted by 19 physician and 34 nurses per 10,000 population [39]. This study suggests that the death rate of Indonesian HCWs was higher than in United States (0.49 deaths per 1000 HCWs), Brazil (0.46) and India (0.25), but lower than in Peru (8.48) and Mexico (3.8) [40–44].

Observational retrospective studies are required to estimate the number of infections and excess deaths attributable to COVID-19 among HCWs. However, in Indonesia, a credible estimate of HCWs mortality amounted to all SARS-CoV-2 infections was unavailable. According to a study of 1,201 specimens collected from HCWs in Jakarta province during in early pandemic, 7.9% of those samples were confirmed positive for SARS-CoV-2. The majority of positive specimens were obtained from medical doctors and nurses (44.2%) [45]. Most of them (64%) reported having contact with suspect/confirmed COVID-19 cases. In the early stages of a pandemic, the screening rate among HCWs remains low [45]. The number of reported deaths due to COVID-19 may be lower than the actual deaths. Indonesia only counted and reported deaths with molecular confirmation and neglected counting deaths without molecular testing (probable death). The source of the problem may come from variable capacities of testing and tracking among provinces.

Investment in the national surveillance system and sharing data of infections and deaths are important for the implementation of preventive actions. Similar issues have arisen in the United States, Italy, and the United Kingdom, where data were limited and challenging to obtain [46]. Essential data on socio-demographic, occupational type, working place, and vaccination status remained difficult to collect from official sources. In lieu of this, we collaborated with the journalist’s network and volunteers to fill the data gap by curating content from social media and contacting medical and healthcare organizations.

Moreover, financial compensation for the deceased’s family is essential to support the livelihood of families. The Indonesian government regulated legal basis of financial compensation for those HCWs who cared for COVID-19 patients in hospitals and/or died due to COVID-19 infections [47]. By end of July 2021, only 218 families of deceased HCWs confirmed receiving the financial compensation. Thus, community groups and medical and healthcare associations worked on advocacy and reimbursement to ensure that all families receive it [48].

Our study has limitations within necessitate careful interpretation of our findings. First, individual data were obtained from *Pusara Digital* records which lacked of 35% of age information and 48% of vaccination status information. Second, data on the age and gender distribution of HCWs in Indonesia are unavailable to public. Consequently, we were unable to calculate standardized mortality rate by age and gender to improve the interpretation of the relative risk compared to the general population at risk from COVID-19.

Finally, this study suggests that the COVID-19 pandemic confirms the high priority need of robust healthcare systems against any future emerging pandemic threats. Further advancement in molecular diagnostic research and technology, rapid community surveillance and epidemiological and clinical studies, integrated data management, data transparency and data analysis, information technology for supply chain, trainings, communication, workforce management is crucial for preventing and responding to the next epidemic.

## Conclusion

This study finds that the COVID-19 event in Indonesia resulted in the loss of many hundreds of HCW, most of them being senior physicians, nurses, and midwives. They died at rates 5-times higher than everyone else. The sheer sparseness of the workforce requires more protective steps and a national systematic surveillance of occupational mortality is urgently needed in this setting.
